# Supplementary material for: Posttranscriptional Regulation in Response to Different Environmental Stresses in Campylobacter jejuni
Source: Microbiol Spectr. 2022 Jun 9;10(3):e00203-22. doi: 10.1128/spectrum.00203-22 (PMC9241687; doi:10.1128/spectrum.00203-22)
Supplement: SUPPLEMENTAL FILE 1 — Supplemental material. Download spectrum.00203-22-s0001.pdf, PDF file, 1.2 MB [file spectrum.00203-22-s0001.pdf]

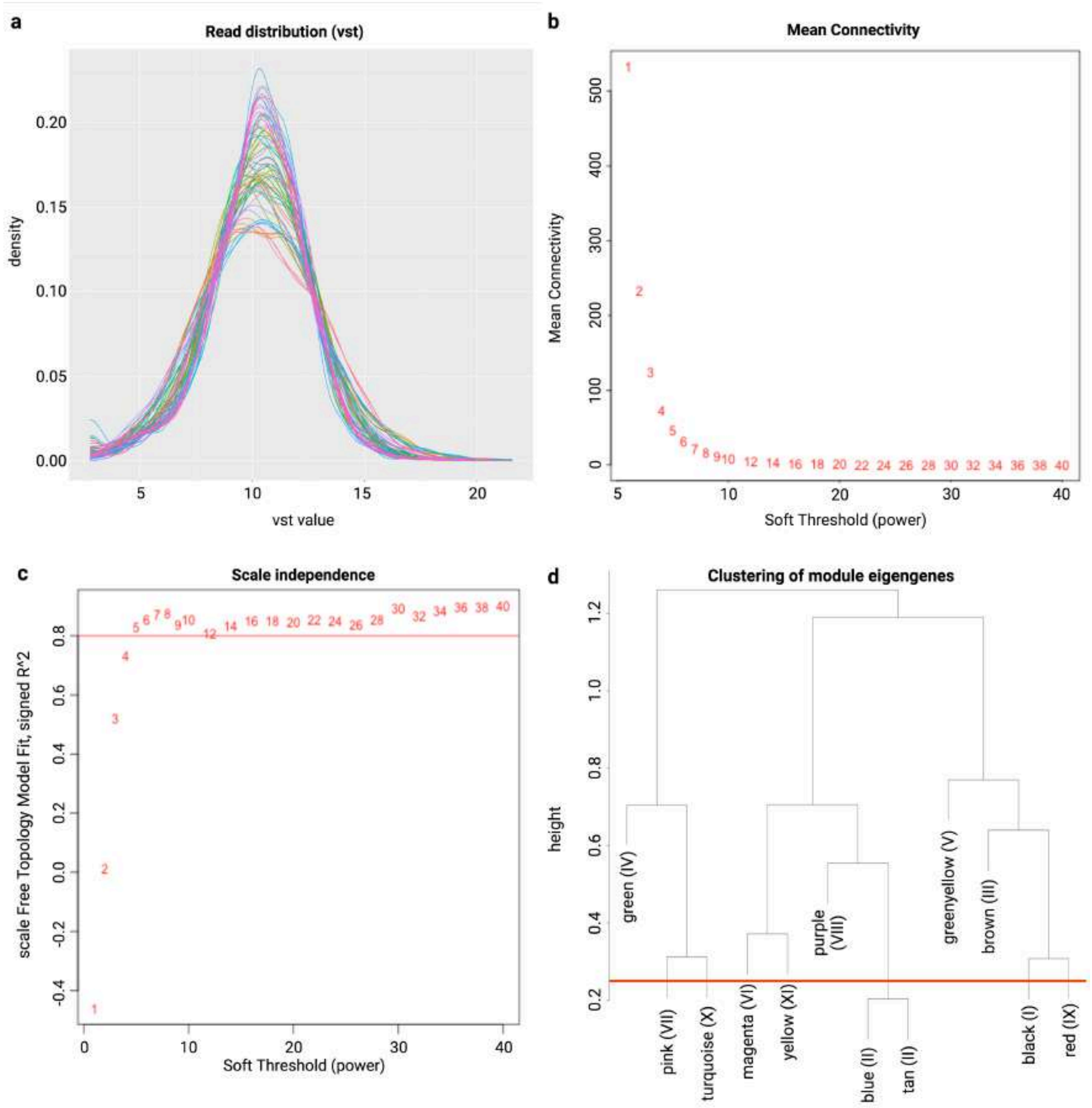

Figure S1: (a) vst transformation normalised the library distribution of all 63 replicates. (b) Mean connectivity of different levels of soft-threshold power transformation (c) scale-fit topology fit of different levels of soft-threshold power transformation (d) Dendrogram of 11 co-expression modules eigengenes. The modules were assigned as different colours by WGCNA. Those colour names were manually replaced using I - XI (see the roman characters inside the bracket. The red line indicates the threshold for dynamic tree cut (0.25).

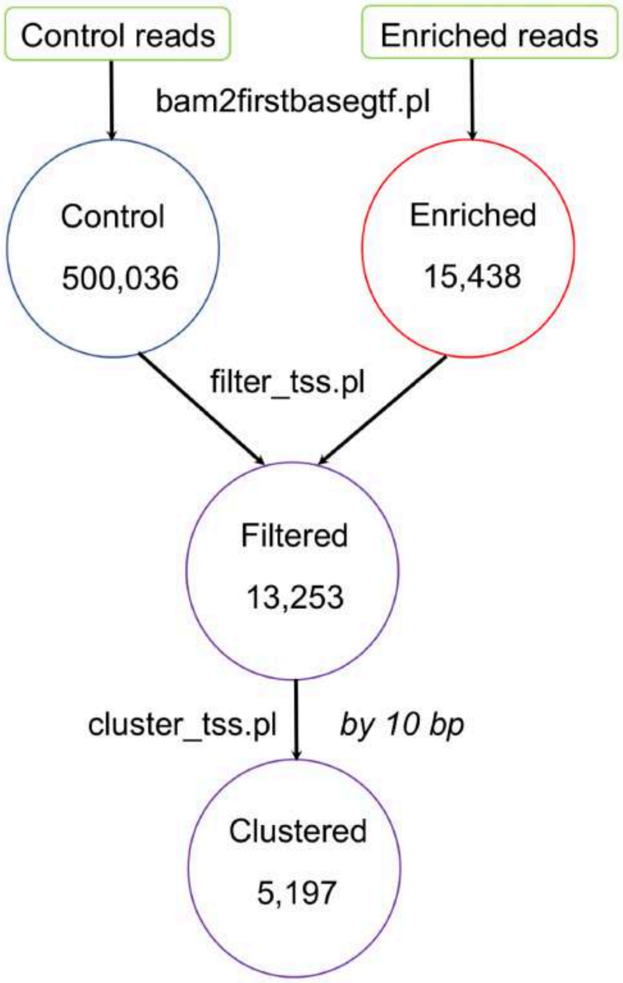

Figure S2: Workflow of Cappable-seq data analysis, which includes filtering false-positive discoveries with streptavidin-free control sample and clustering proximal TSS. The numbers in the circles indicate the number of TSS.

Table S1: Number of Cappable-seq identified TSS that are novel, enriched and/or associated to a consensus promoter

**TSS type**	**Total**	**Novel**	**Novel and enriched**	**Novel, enriched, with promoter**
Primary	637	154	154	123
Secondary	317	192	176	143
Internal	2956	2388	2277	1949
Antisense	1257	861	855	767
Orphan	30	18	17	14
Total	5197	3613	3479	2996

Table S2: All northern blot validated sRNAs from previous publications and their corresponding predicted sRNAs

validated sRNA	predicted sRNA
CjNC9	CjSA4
SRP RNA	CjSA9
CJnc10	CjSA14
CJnc20	CjSA22
RnpB	CjSA51
CJnc60	CjSA64
CJnc110	CjSA88
CJnc120	CjSA90
6S RNA (CJnc130)	CjSA91
CJnc140	CjSA93
tmRNA	CjSA97
crRNA2	CjSA103
crRNA4	CjSA103
TracrRNA	CjSA103
CJnc170	CjSA108
CJnc180	CjSA109
CJnc190	CjSA110
CJnc230	CjSA116
CJas_Cj1667c	CjSA112

Table S3: All pairwise comparision

sample	control	stress conditions
37_ES	37_M	growth phase
37_LS	37_M	growth phase
37_LS	37_ES	growth phase
5%_ce	37_M	temperature
acid	37_M	acid
ana	37_M	anaerobic
cold	37_M	temperature
GSNO	37_M	nitrosative
heat	37_M	temperature
nacl	37_M	hyperosmotic
oxidative	37_M	oxidative
starv	37_ES	starvation
42_ES	42_M	growth phase
42_LS	42_M	growth phase
42_LS	42_ES	growth phase
42_M	37_M	temperature
42_ES	37_ES	temperature
42_LS	37_LS	temperature
iron_lim_ES	iron_rep_ES	iron limitation
iron_lim_M	iron_rep_M	iron limitation
iron_lim_ES	iron_lim_M	growth phase
iron_rep_ES	iron_rep_M	growth phase
sod_deoxy_ES	37_ES	bile salt
sod_deoxy_M	37_M	bile salt
sod_deoxy_ES	sod_deoxy_M	growth phase

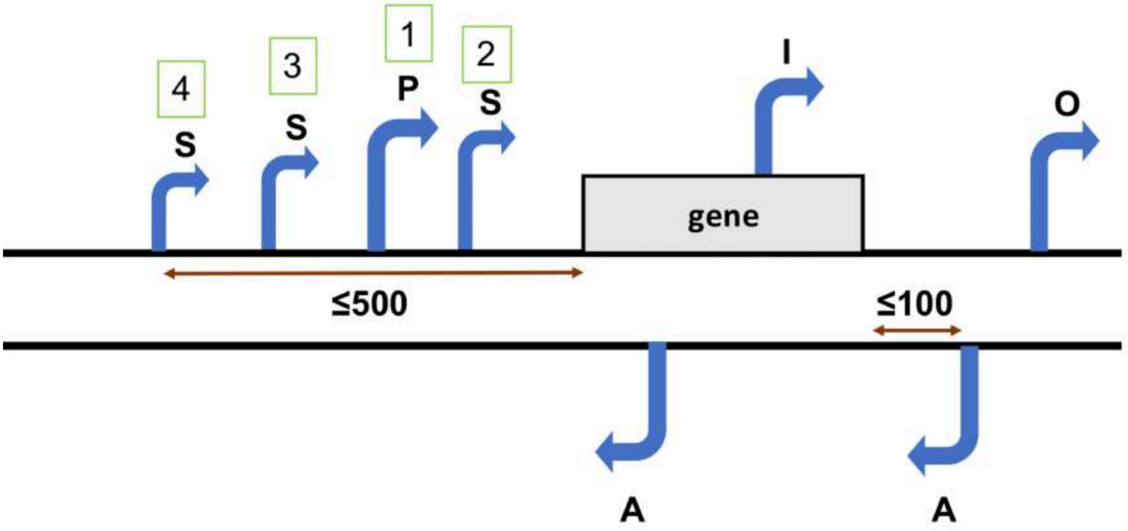

Figure S3: Categorisation of TSS. P = Primary, S = Secondary, I = Internal, A = Antisense, O = Orphan.

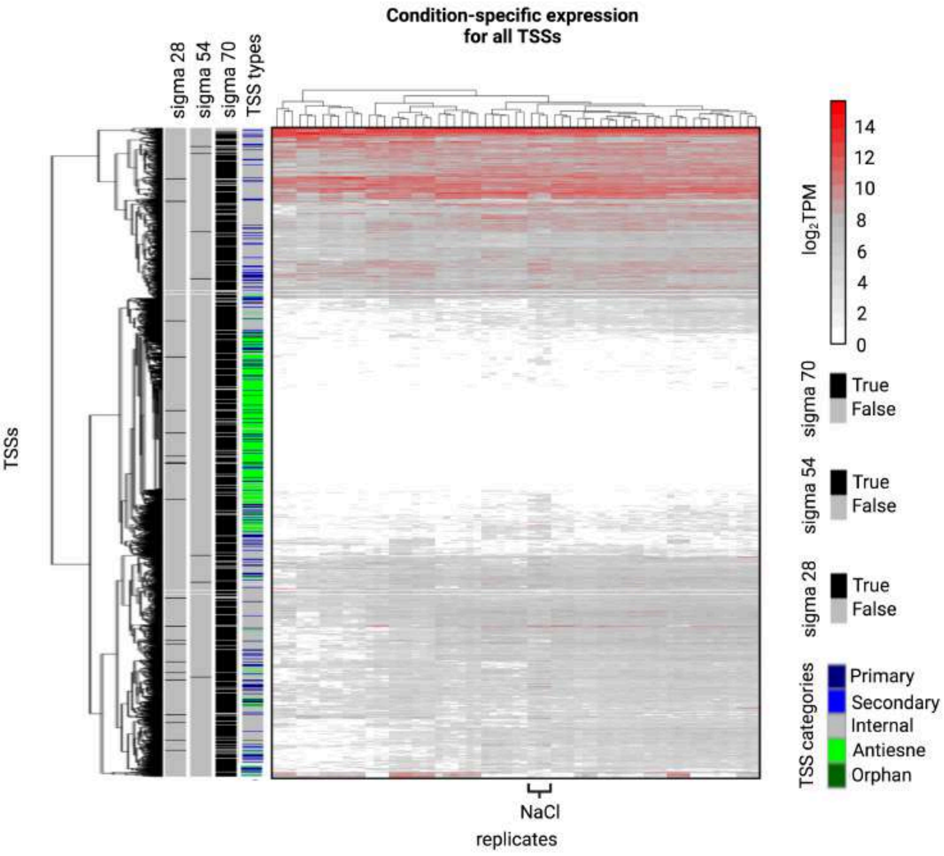

Figure S4: $\log_2$TPM expression of all Cappable-seq. All TPM values were determined using the expression from the 10 bp upstream of each TSS. If a TSS has a TPM value of 0, its corresponding $\log_2$TPM was transformed to 0.

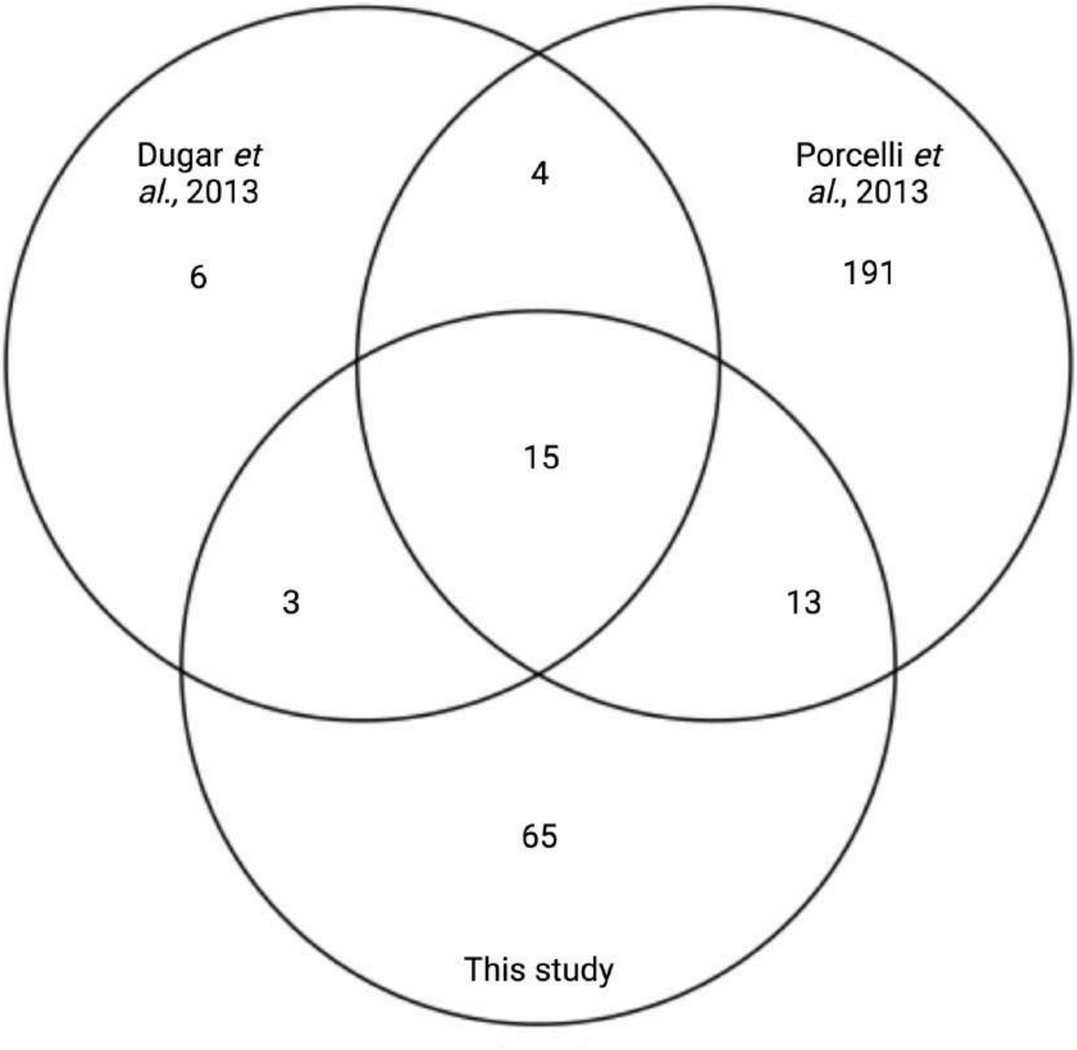

Figure S5: Comparisons of sRNA predicted from this study to sRNA (either detected by dRNA-seq, validated by northern blot, or both) from Dugar et al.,2013 and Porcelli et al.,2013.

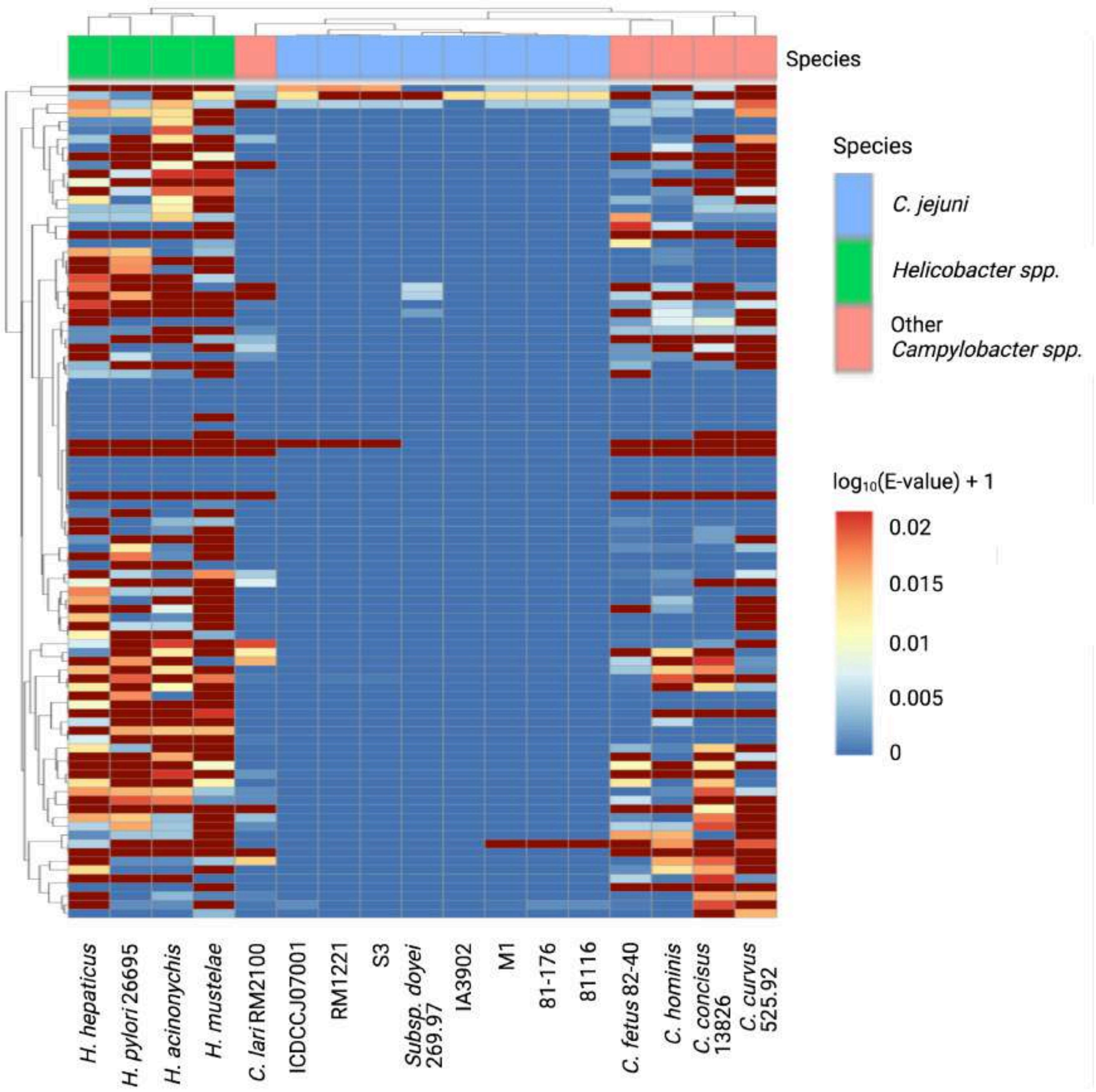

Figure S6: Conservation of all predicted sRNAs was compared using BLASTN E-values sRNAs against representative Epsilonproteobacteriastrains. Lower E-values indicate better sequence alignment. Queries without any homologous alignments were depicted with dark red.

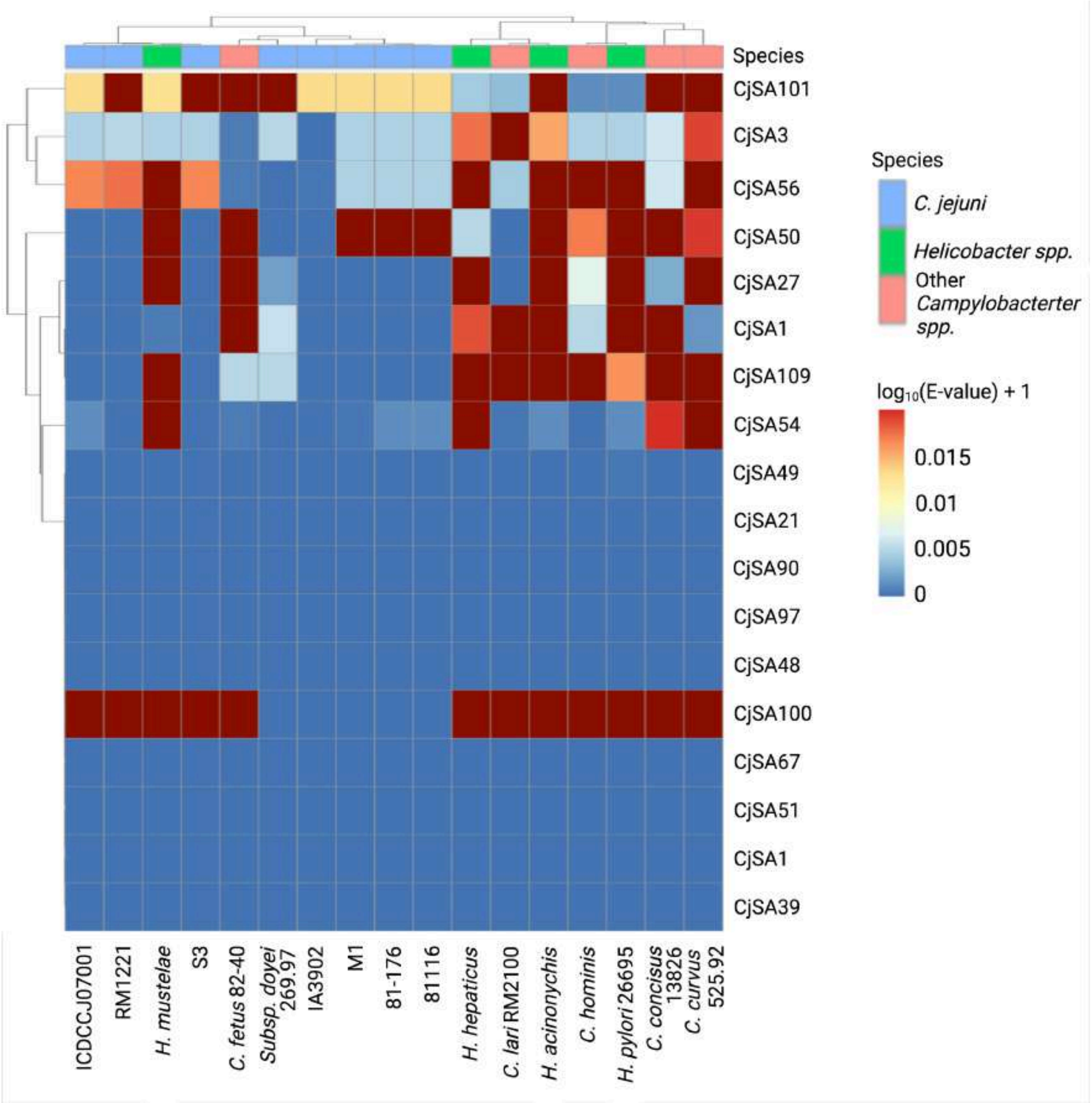

Figure S7: BLASTN output of predicted sRNAs conserved among all Epsilonproteobacteria strains or not/less conserved in at least one *C. jejuni* strain. Queries without any homologous alignments were depicted with dark red.

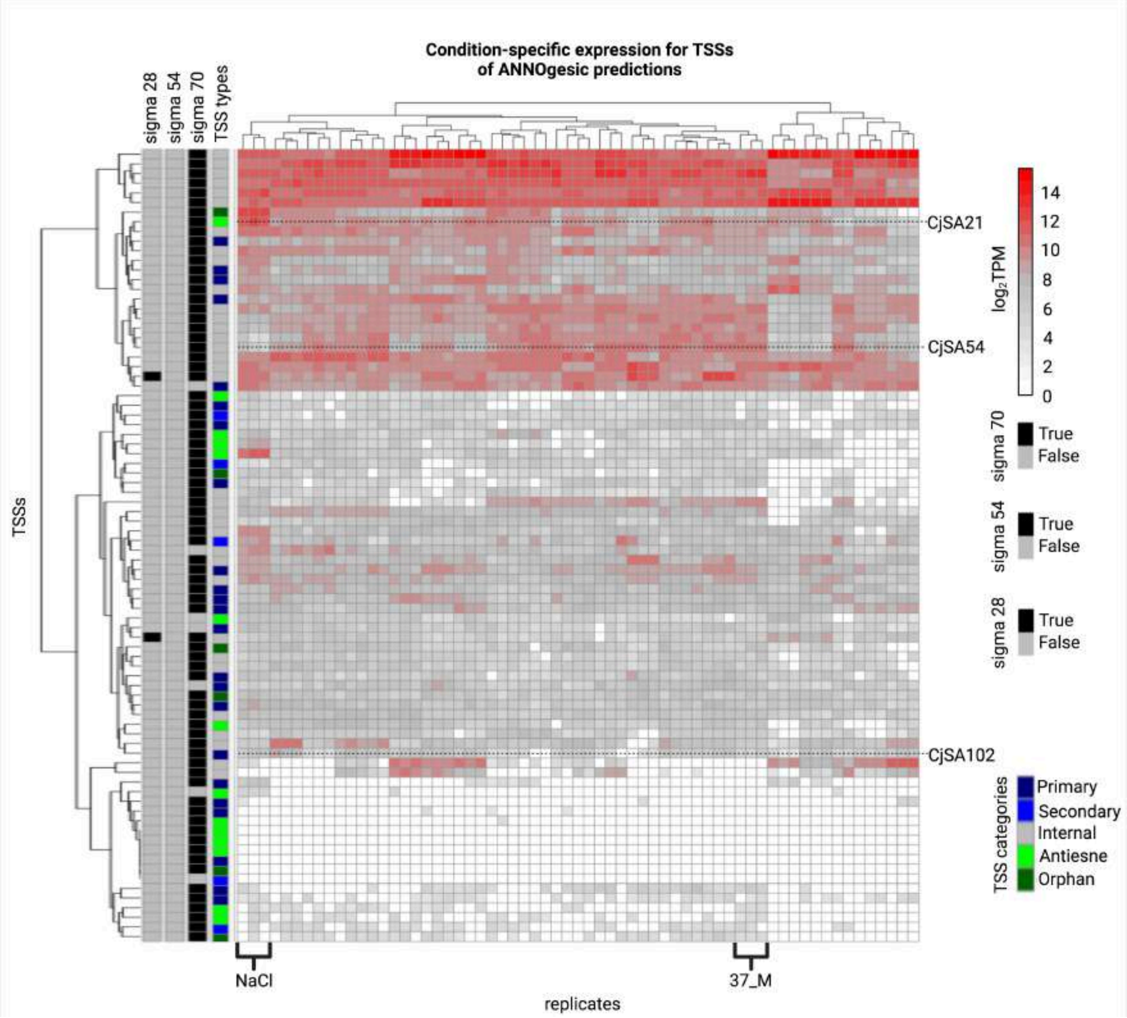

Figure S8: $\log_2$TPM expression of those Cappable-seq TSS that regulate ANNOgesic predicted sRNAs. All TPM values were determined using the expression from the 10 bp upstream of each TSS. If a TSS has a TPM value of 0, its corresponding $\log_2$TPM was transformed to 0.

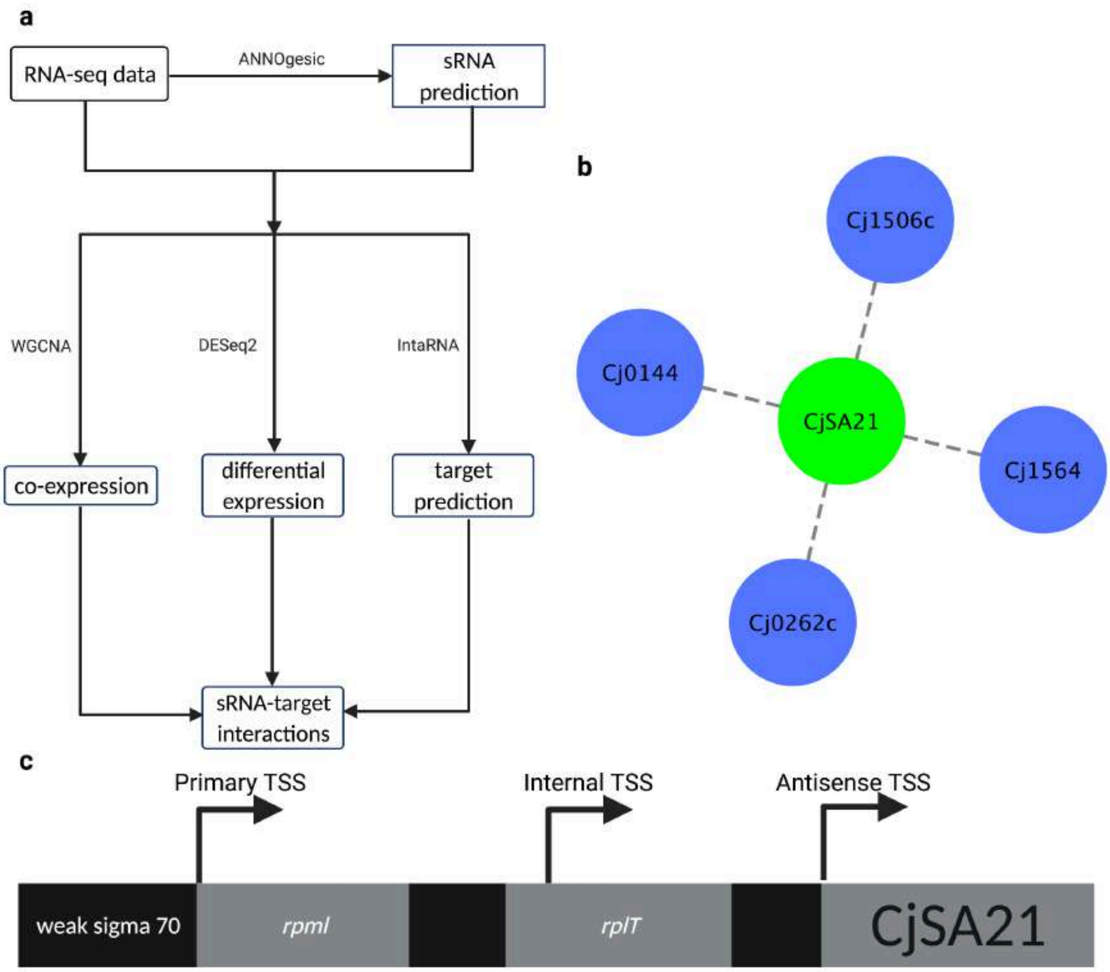

Figure S9: (a) The predicted sRNA and RNAtag-seq data were processed by integrating co-expression analysis, differential expression analysis and genome-wide sRNA-target prediction. The integrative analysis aims to uncover sRNA-target interactions with the following criteria: 1. Both genes must belong to the same WGCNA co-expression modules and are directly connected by the WGCNA network. The correlation coefficient between two gene expressions must be statistically significant (FDR $\leq$ 0.05). 2. Two genes need to show enough desired expression patterns that agree with the co-expression analysis. In other words, two genes with negative co-expression need to show opposite differential expression. If one gene is differentially upregulated in one condition, the other one must be differentially downregulated in the same condition. The number of conditions with desired differential expression patterns needs to be at least two more than the number of conditions with undesired differential expression patterns 3. Stable optimal binding energy calculated by IntaRNA, with p-value $\leq$ 0.05. (b) Highlighted sRNA-target interaction involving CjSA21. The dashed line indicated negative co-expression (c) Genome organisation of CjSA21 and transcription start sites and open reading frames in the proximity. Created in BioRender.com

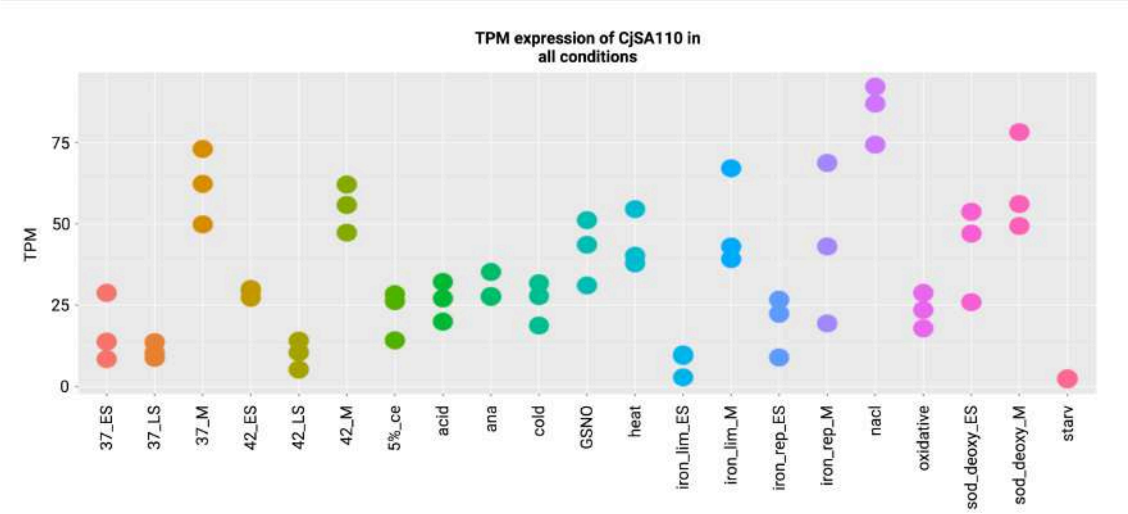

Figure S10: TPM normalised expression of CjSA110 across all 21 experimental conditions.

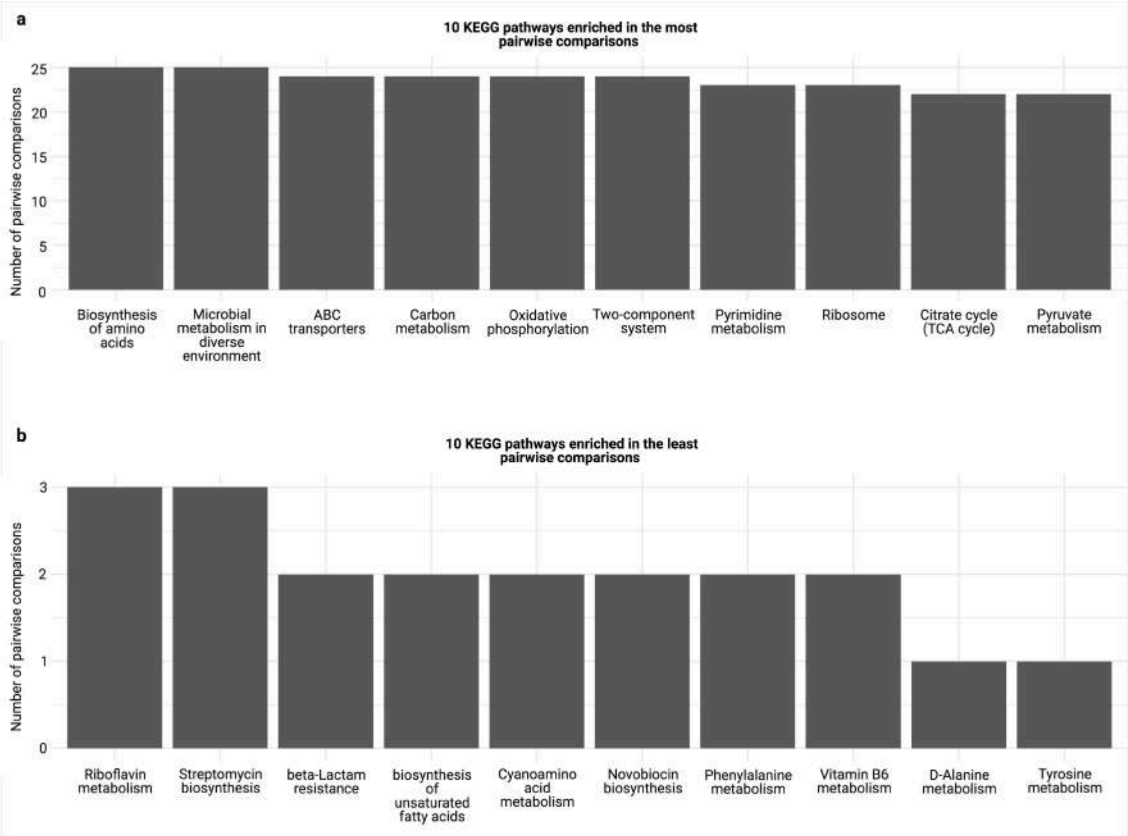

Figure S11: (a) The 10 KEGG pathways that are statistically overrepresented in the most number of DESeq pairwise conditions. (b) The 10 KEGG pathways that are statistically overrepresented in the least number of DESeq pairwise conditions.

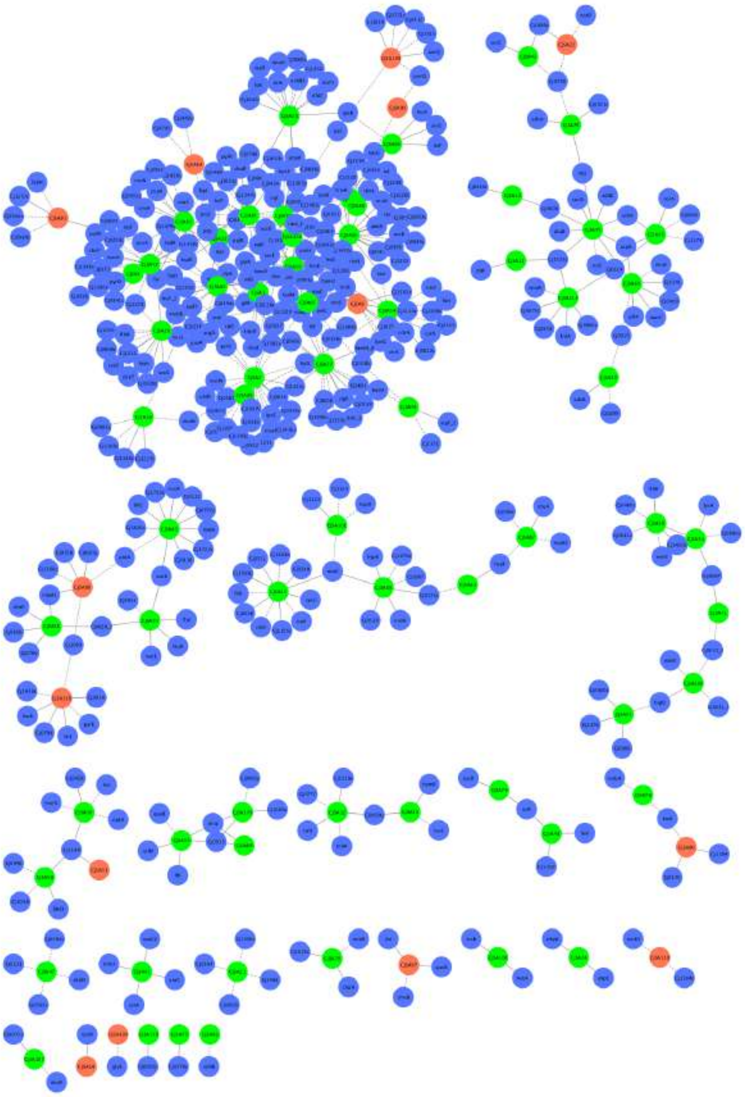

Figure S12: Cytoscape visualisation with all high-confidence sRNA-target interactions. Dashed lines represent a negative correlation, while solid lines represent a positive correlation. All novel sRNA nodes are coloured in green, while those that correspond to published sRNA are coloured in orange (default = purple).

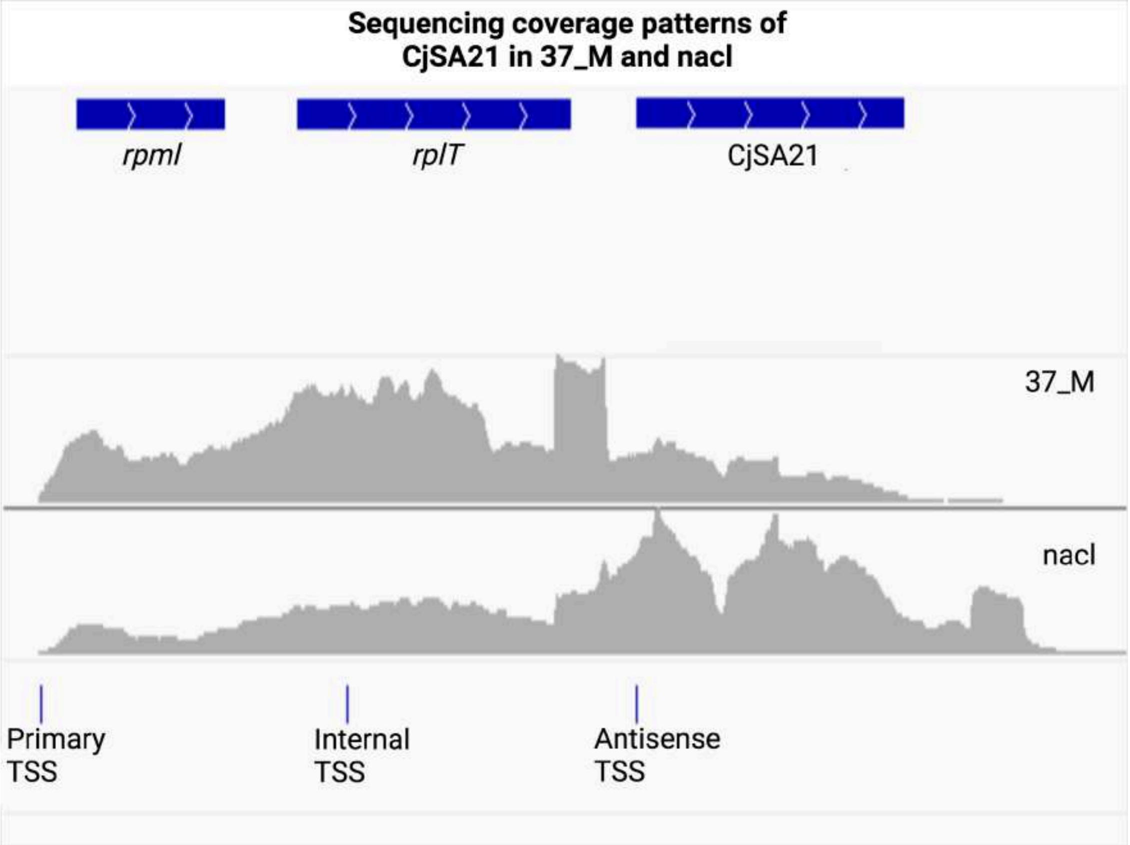

Figure S13: Sequencing coverage patterns across the CjSA21 promoter region in 37_M and nacl. Only the sense strand coverage is shown here.

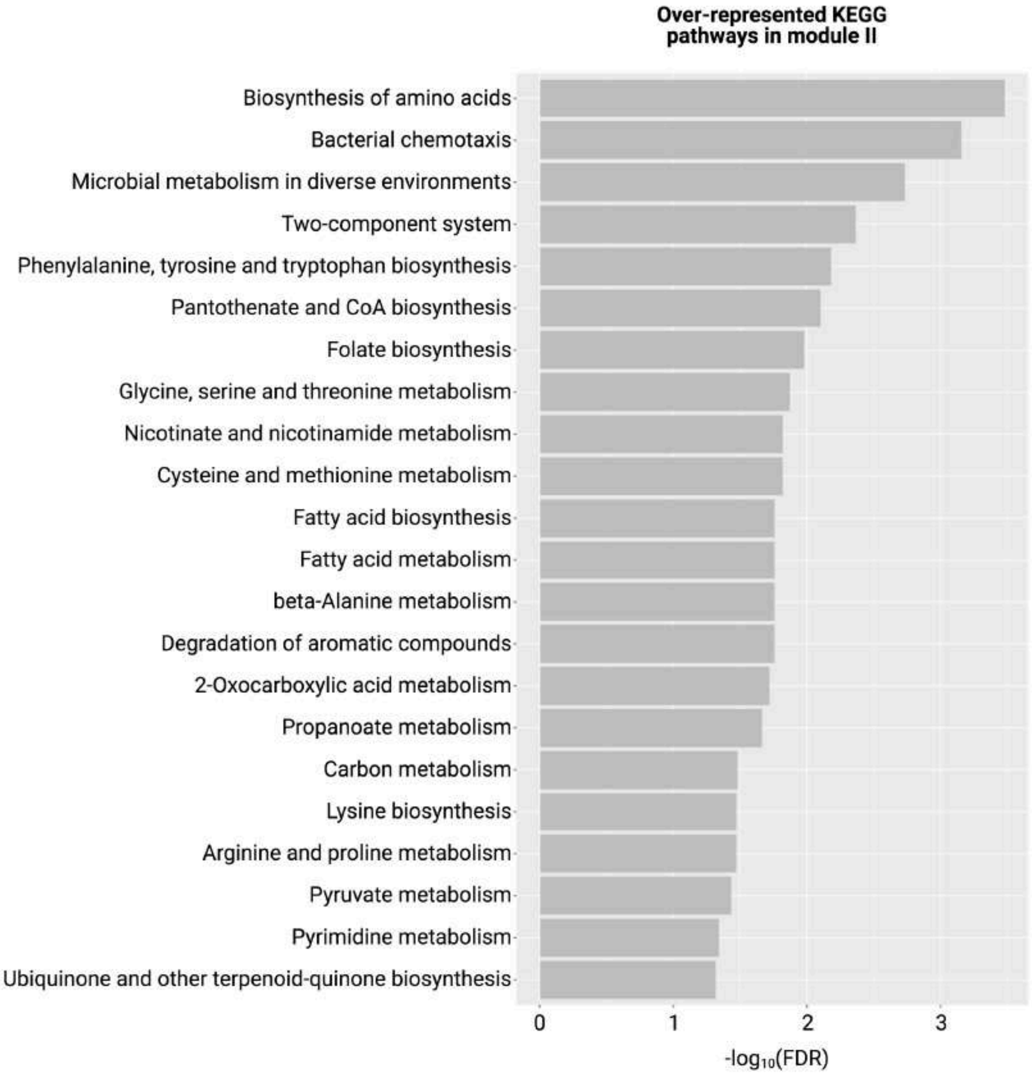

Figure S14: All enriched KEGG Pathways (FDR ≤ 0.05) of module II. Only pathways with less than 100 proteins are included
.

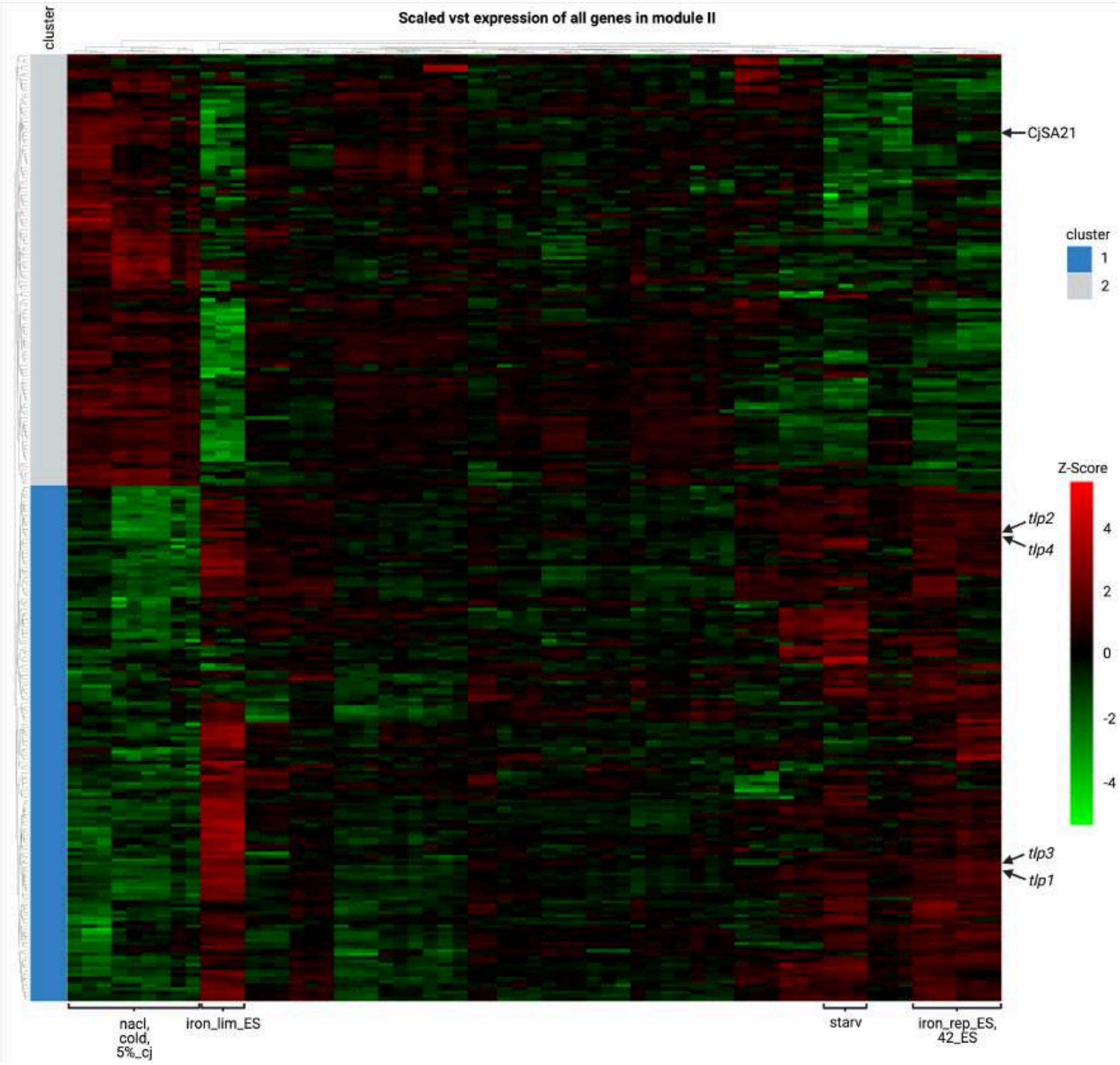

Figure S15: The scaled vst score and complete linkage hierarchical clustering of all genes in module II. The heatmap is scaled by rows.

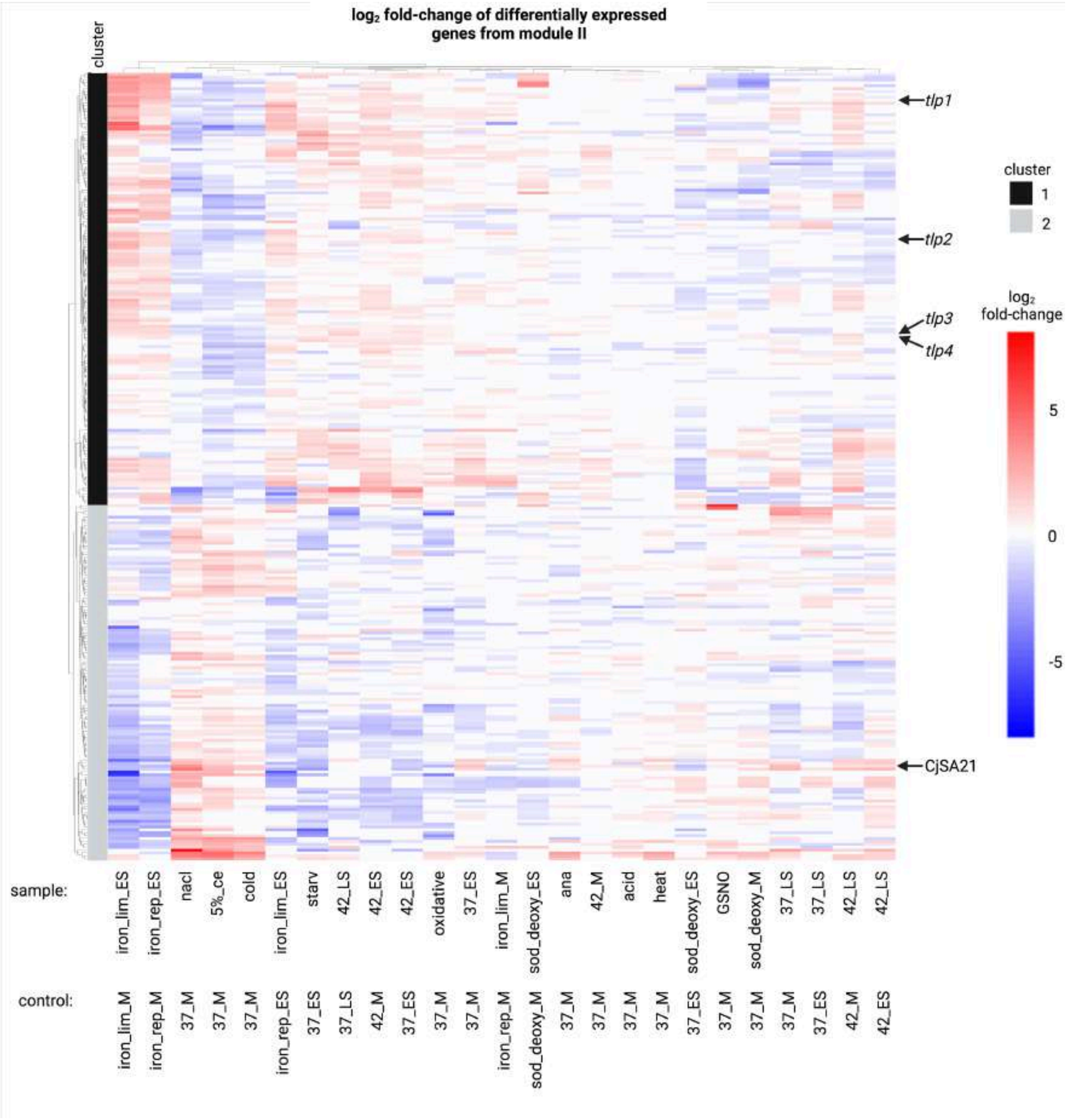

Figure S16: The $\log_2$ fold-change and complete linkage hierarchical clustering of all genes in module II. All boxes with person correlation p-value > 0.05 are coloured as white.

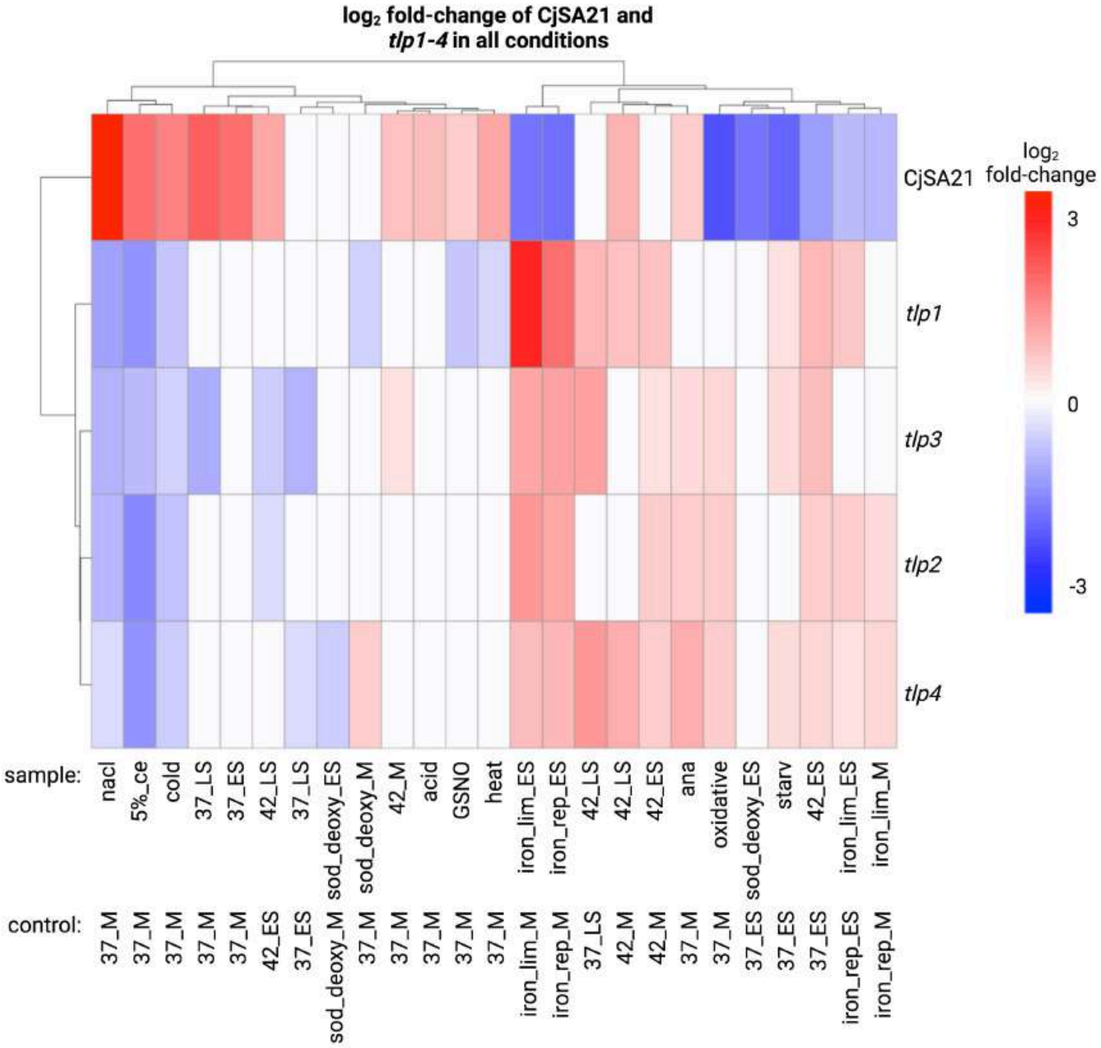

Figure S17: $\log_2$ fold-change of CjSA21 and *tlp*1-4 across 21 experimental conditions. Genes with FDR above 0.05 are coloured as white.

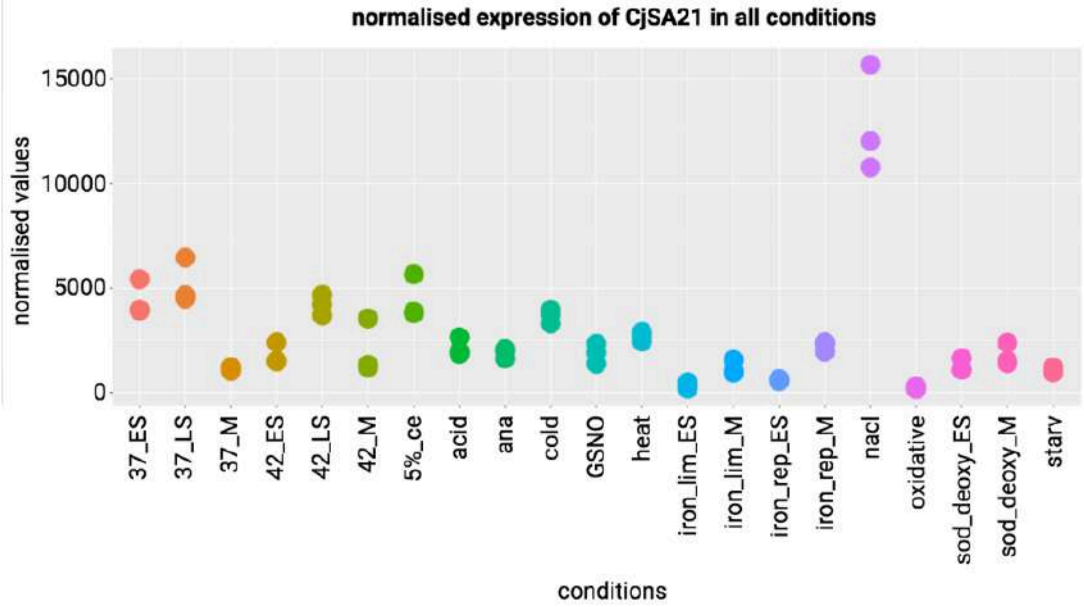

Figure S18: Expression levels of CjSA21 across 21 experimental conditions. The expression value was normalised by DESeq's median to ratio.

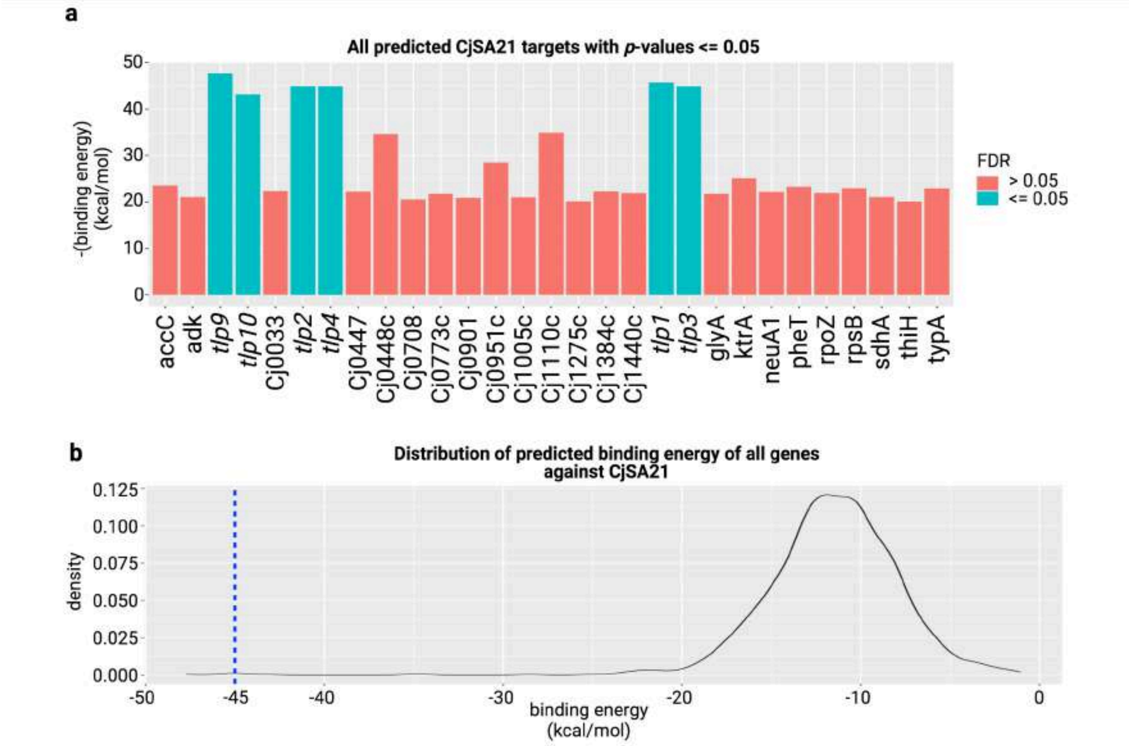

Figure S19: (a) Computed binding energy of all targets with p-values ≤ 0.05. Those bars coloured in light blue has FDR ≤ 0.05. (b) IntaRNA binding energy distribution of all sRNA-mRNA pairs. The blue line indicates -45 kcal/mol, which is approximately the predicted binding energy values against *tlp1-4*

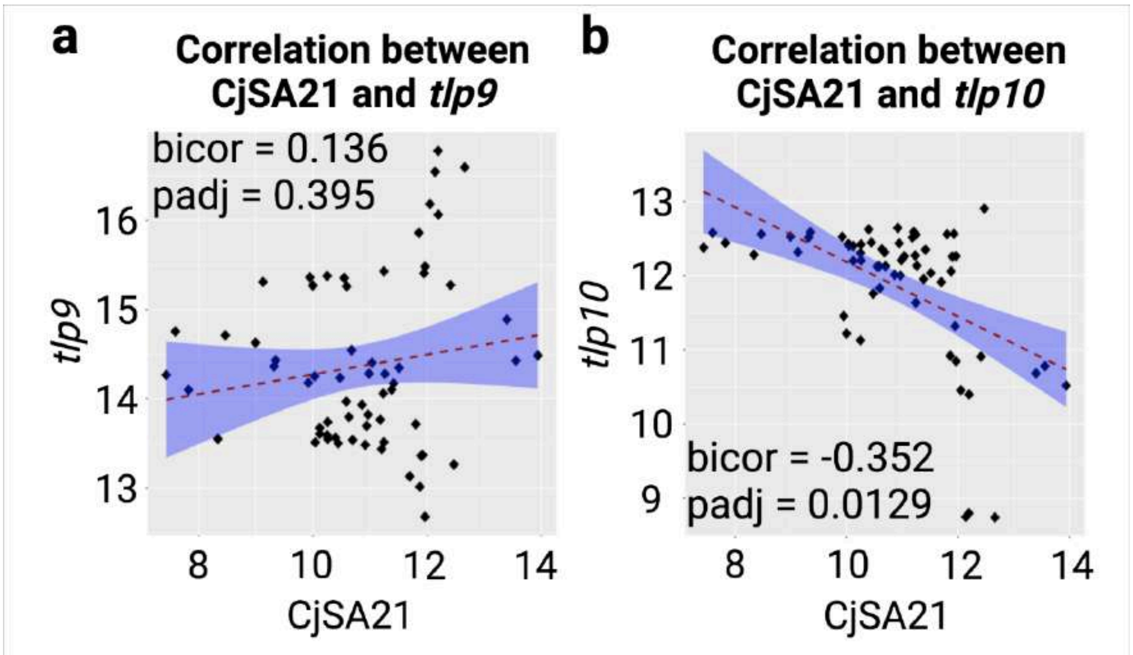

Figure S20: Pairwise comparisons expresssion of CjSA21 against (a) *tlp9*, (b) *tlp10*.

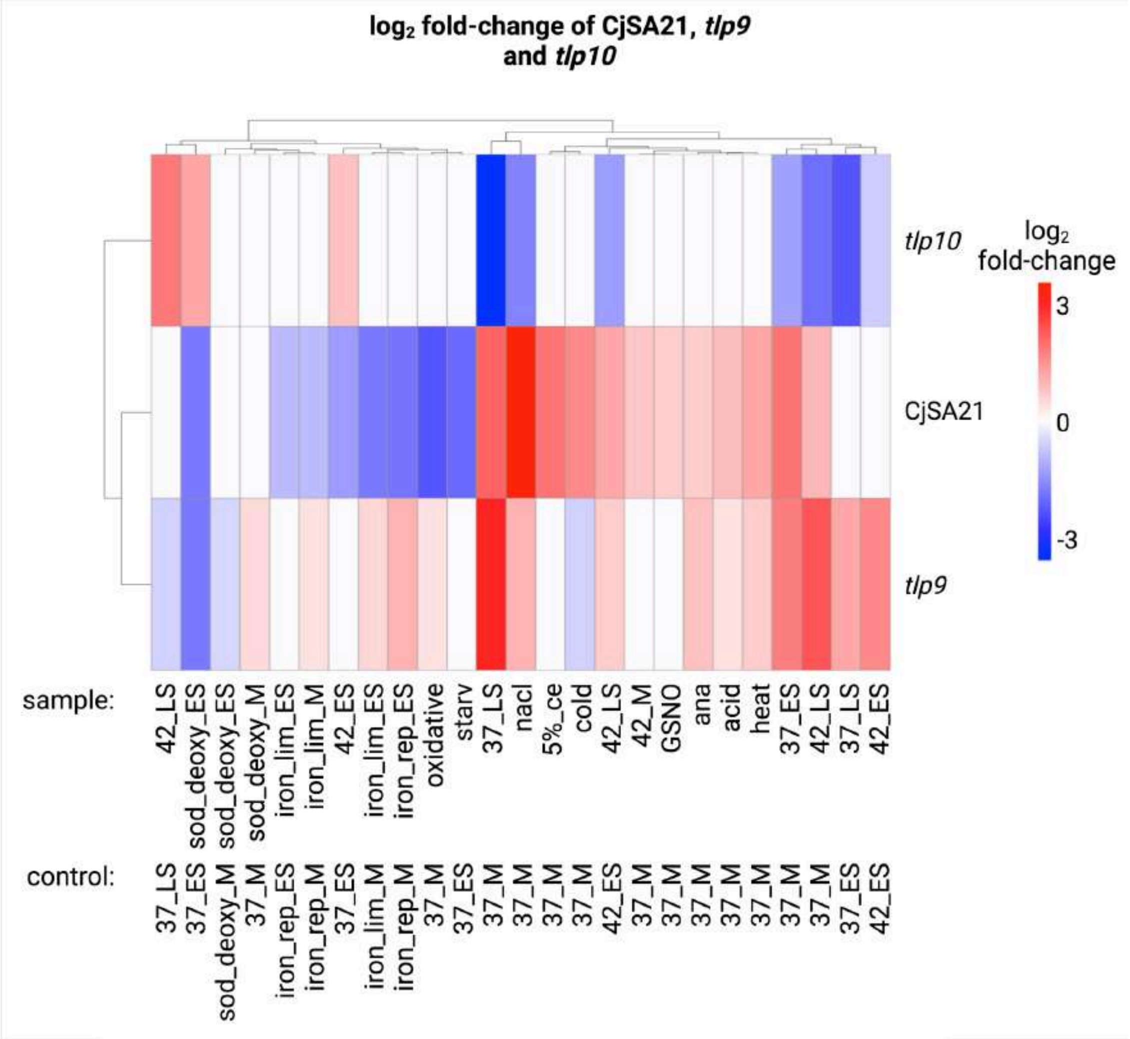

Figure S21: $\log_2$ fold-change of CjSA21, *tlp9* and *tlp10* across all selected pariwise comparisons. Those data with padj > 0.05 are coloured in white.

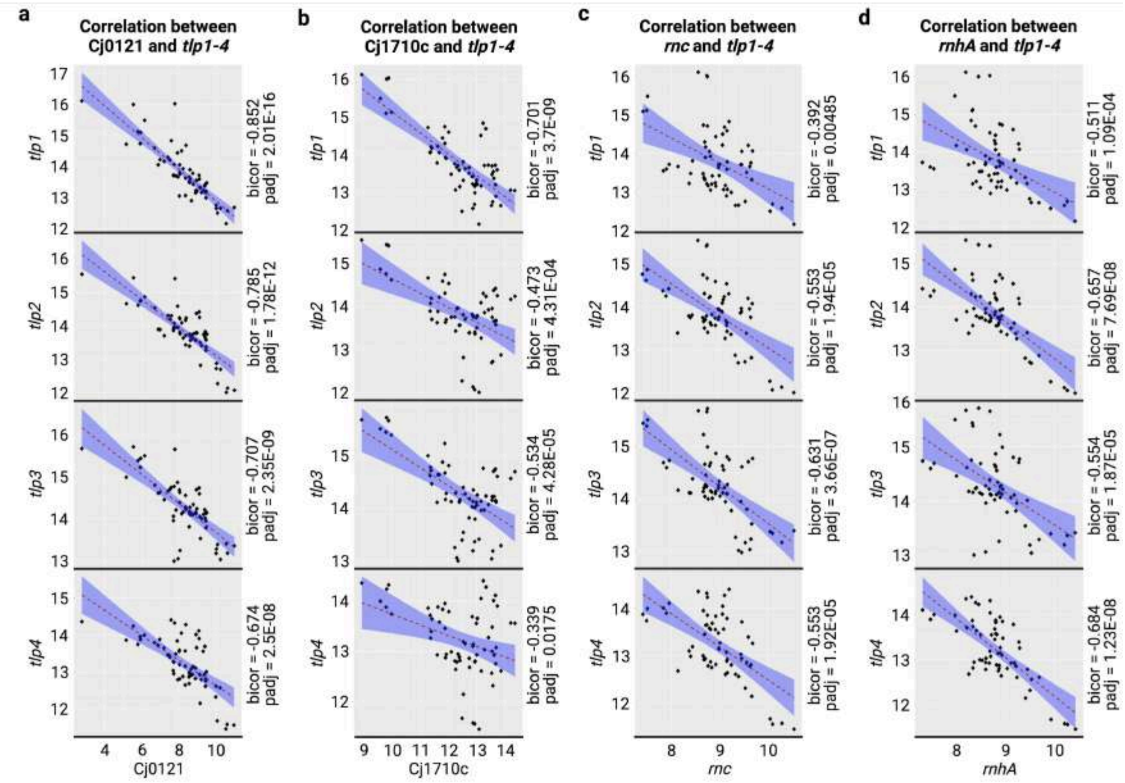

Figure S22: Pairwise comparisons of vst values of *tlp1-4* against RNases in module II, which includes (a) Cj0121, (b) Cj1710c (c) *rnc* and (d) *rnhA*.

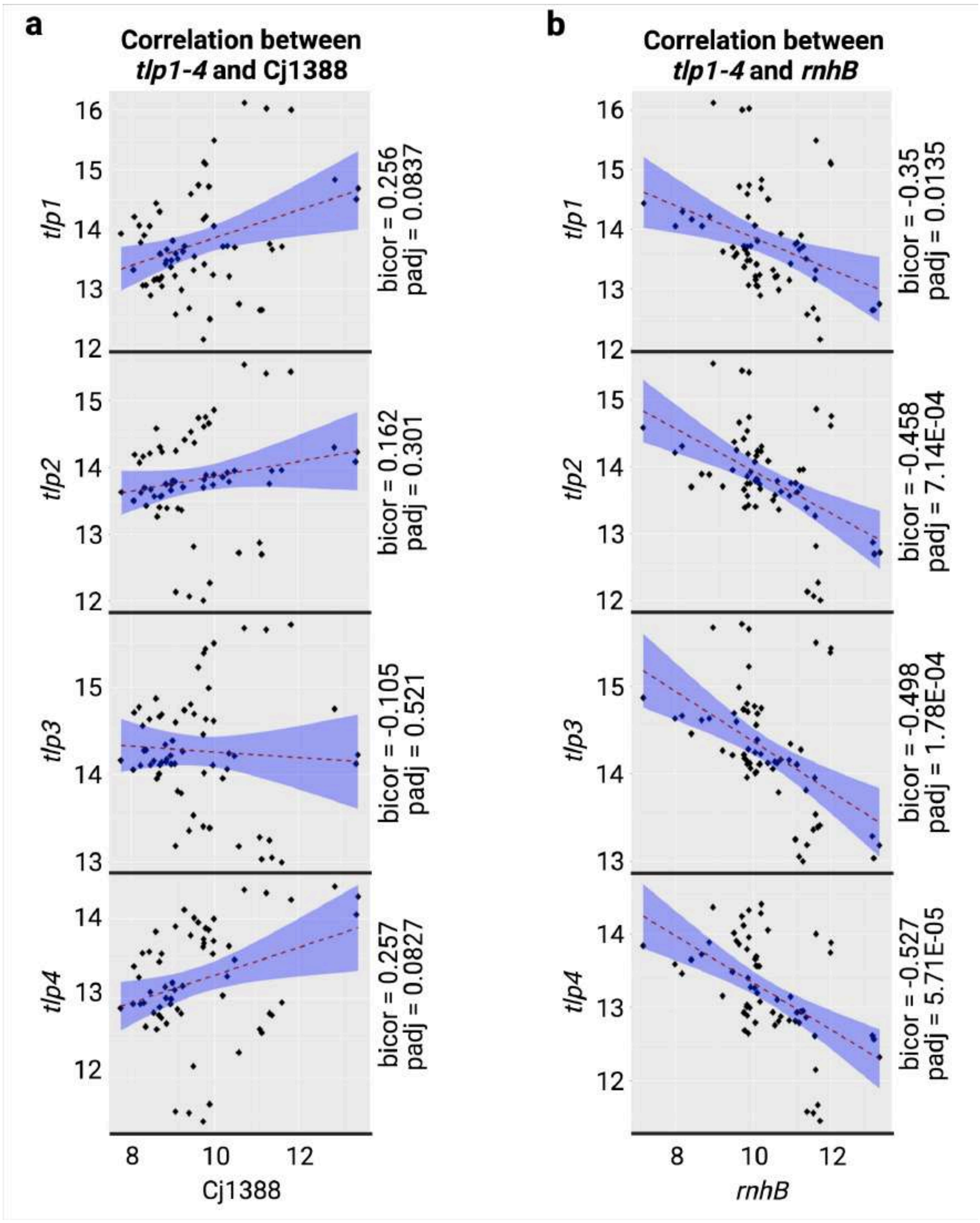

Figure S23: Pairwise comparisons expresssion between *tlp1-4* against (a) Cj1388 and (b) *rnhB*.

Details of experimental conditions

Growing NCTC11168 without supplements

Unless specified otherwise, *C. jejuni* NCTC11168 growth was cultured in MH2 cation-adjusted broth (Sigma) in the plate reader at 37 °C. The growth conditions are detailed in Table 1.

Table 1: All growth conditions under 37 °C.

**Condition**	**Growth phase**	**OD_{600}**	**Time (hours)**
37_M	exponential	0.765-0.9	10
37_ES	early stationary	1.575 or above	21
37_LS	late stationary	before declining	38

For conditions involve cell growth at 42 °C (42_M, 42_ES, 42_LS), the cells were cultured in the same setting as 37 °. The growth optical density and duration are shown in Table 2.

Table 2: All growth conditions under 42 °C.

**Condition**	**Growth phase**	**OD_{600}**	**Time (hours)**
42_M	exponential	0.675-0.81	7
42_ES	early stationary	1.58-1.71 or above	16
42_LS	late stationary	before declining	26

Growing NCTC11168 with supplements

Bile stress was modelled by supplementing the MH2 media with 0.1 % sodium deoxycholate to mimic growth under bile salt. The cells were harvested at either exponential or early stationary phases. See Table 4 for more details.

Table 3: All growth conditions under 0.1 % sodium deoxycholate.

**Condition**	**Growth phase**	**OD_{600}**	**Time (hours)**
sod_deoxy_M	exponential	0.076-0.135	6
sod_deoxy_ES	early stationary	0.27-0.34	12

Iron-limited conditions (iron_lim_M and iron_lim_ES) were modelled by replacing with MH2 broth with MEMα (Gibco) supplemented with 10 µM pyruvate. Iron repleted conditions (iron_rep_M and iron_rep_ES) were cultured in the same setting as iron-limited conditions, with the addition of $FeSO_4$ in the media. Both iron-limited and iron-repleted conditions were

harvested at exponential and early stationary phases. See Table **??** for more details.

Table 4: All growth conditions under iron-repleted and iron-limited conditions.

**Condition**	**Growth phase**	**OD_{600}**	**Time (hours)**
iron_lim_M	exponential	0.135-0.225	4.5
iron_lim_ES	early stationary	0.225-0.27	12
iron_rep_M	exponential	0.135-0.225	4.5
iron_rep_ES	early stationary	0.72-0.765	12

Hyperosmotic (nacl) and nitrosative (GSNO) stress

C. jejuni cells were cultured in the same way as 37_M. Hyperosmotic stress was applied by re-suspending cell cultures in a 1.5 % NaCl MH2 broth filter that was sterilised with 0.22 µM pore membrane filters (Millipore). Nitrosative stress involves adding 1.5 mM of S-Nitrosoglutathione (GSNO) (CALBIOCHEM) to the cultures. Before applying stresses, cells were grown to OD_{600} 0.45-0.675 to allow extra 2 hours of incubation in the plate reader.

Starvation stress (starv)

After growing under the same condition as 37_ES, NCTC11168 cells were centrifugated for 3 minutes at 5000 x g. The cell pellets were resuspended in sterile Ringer's solution (2.25 g/L NaCl, 0.08 g/L $CaCl_2.2H_2O$, 0.05 g/L $NaHCO_3$) in a 6-well plate (Greiner), followed by a 5 hours incubation in the plate reader.

Acid shock (acid)

After growing the cells at the same condition at 37_M and centrifuging for 3 minutes at 5000 x g, the cell pellet was re-suspended at MH2 broth calibrated to pH 3.5. The MH2 broth was calibrated using 30 % Hydrochloric acid (Fisher Scientific) and filter sterilised with 0.22 μM pore membrane filters (Millipore).

Anaerobic (ana) and heat (heat) stress

The *C. jejuni* culture was first grown under 37 °C to the exponential phase. Afterwards, the cell 11168 culture was incubated for 1 hour in a 55 °C incubator at standard atmospheric conditions for 3 minutes for heat shock or in an anaerobic VAIN (80 % N2, 10 % CO2, 10 % H2) for anaerobic stress.

Oxidative stress (oxi)

After reaching the exponential phase at 37 °C, 3.5 ml of cell culture was supplemented with 1.19 muL of hydrogen peroxide (30% w/v) (Fisher Scientific), leading to a final hydrogen peroxide concentration of 3 mM. The supplemented culture was further incubated for 10 minutes.

Chicken exudate (5% ce) and cold stress (cold)

The chicken exudate was extracted from a whole frozen chicken was purchased from Waitrose, UK. The chicken was defrosted overnight at 4 °C and then at room temperature for a few hours. After defrosting, the chicken exudate was collected in 50 mL falcon tubes and filter sterilised using 0.22 μM pore membrane filters (Millipore). The chicken exudate was stored at -20 °C up to one month until further use.

An AnaeroJarTM (Oxoid) of 2.5 L was reduced with a CampyGen sachet (Thermo Scientific) to a microaerophilic environment. After growing under the same condition as 37_M, 3.5 mL of NCTC 11168 culture was centrifugated at 5000 x g for 3 minutes. The cell was then re-suspended in the same volume of diluted chicken juice (5 % v/v) in 25 cm^3 Vented Capped Tissue culture flasks (Falcon). Following the resuspension, cells were incubated in the microaerophilic AnaeroJar for 24 hours at 4 °C. After the incubation, cells were harvested with cold killing buffer followed by RNA extraction.

The same procedure was carried out without supplementing the MH2 broth with the chicken exudate to model the cold stress.

Details of RNA extraction and processing

RNA extraction

3.5 mL of cell culture was inactivated by adding 0.5 volumes of cold (4 °C) killing buffer (20 mM Tris-HCl (pH 7.5), 5 mM $MgCl_2$, 20 mM NaN_3). Cell cultures were spun down at 5000-10000 x g for 3-5 minutes, then snap frozen in liquid nitrogen and stored at -80 °C until further use.

The pellet was re-suspended in 600 µL of LETS buffer (0.1M LiCl, 0.01M Na_2EDTA, 0.01M Tris-Cl at pH 7.4 and 0.2%SDS) and transferred to Lysing Matrix B tubes (MP biomedicals^{TM}). The tubes were placed in a FastPrep Homogenizer (MP biomedicals^{TM}) to perform three rounds of bead-beating at a speed of 6.0 m/s for 40 seconds, with a 5 minutes pause between rounds. The samples were then centrifuged at 13000 x g for 10 minutes at 4 °C. Afterwards, proteins and nucleic acids were separated from the cell debris by centrifuging at 13000 rpm at 4 °C for 10 minutes. RNA was phase-separated by two rounds of 125:24:1 Phenol/Chloroform/isoamyl alcohol (PCI) and one round of 24:1 chloroform/isoamyl alcohol. The upper phase was mixed with 0.1 volume of 3M sodium acetate (pH 5.2) and 1 volume of isopropanol for overnight precipitation at -20 °C.

After overnight precipitation, samples were spun at top speed at 4 °C for 15 minutes. The supernatant was removed, and pellets were washed (without re-suspending) with fresh 70% ethanol twice. Pellets were air-dried for 15 minutes before re-suspending in 50 µL of RNase-free water. The re-suspended RNA was incubated on ice for three hours, then 30 minutes at room temperature.

RNA samples processing

Genomic DNA was removed from isolated RNA following the TurboDNase (Ambion) manufacturer's protocol. DNase inactivation reagent was not added to avoid downstream effects. To avoid RNA degradation during DNA removal, 1 µL of SUPERase In^{TM} RNase Inhibitor (Invitrogen) was added into every 50 µL of sample. After DNase treatment, RNA samples were purified using RNA Clean and concentrator^{TM} -5 kit (Zymo).

DNA removal was estimated by PCR amplification. The 16S rRNA gene was amplified with bacterial forward primer Bac27F (5'-AGAGTTTGGATCMTGGCTCAG-3') and universal reverse primer Univ1492R (5'-CGGTTACCTTGTTACGACTT-3') using GoTaq Green master mix (Promega).

Afterwards, bacterial rRNA was removed using Ribo-Zero^{TM} rRNA removal kit for bacteria (Illumina, UK). Agilent 2100 Bioanalyzer RNA 6000 Pico kit (Agilent, UK) was used to confirm DNA removal, RNA integrity, and rRNA removal.

Cappable-seq library preparation

RNA samples from all three biological replicates from each condition were normalised and diluted to around 100 ng/µL and pooled together for Cappable-seq processing Ettwiller et al. [2016]. A control sample was used, which omitted the Streptavidin capture step. See Ettwiller et al., 2016 for more details.

The libraries were created from the Cappable-seq processed RNA pool using NEBNext Small RNA Library Prep Set for Illumina. The library was spiked with 5% PhiX (Illumina), and loaded onto a 150 cycle v3 sequencing cartridge (Illumina) and sequenced on Illumina MiSeq single end for 100 cycles (including pre-phasing). The control and Cappable-seq enriched pool were amplified for 12 and 18 PCR cycles, respectively.

RNAtag-seq library preparation

This protocol was adapted from Shishkin et al. 2015. The RNAtag-Seq library was loaded onto a 150 cycle High-Output cartridge and sequenced on the NextSeq 550 system (2x76 cycles paired-end, including pre-phasing).

Bibliography

L. Ettwiller, J. Buswell, et al. A novel enrichment strategy reveals unprecedented number of novel transcription start sites at single base resolution in a model prokaryote and the gut microbiome. *BMC Genomics*, 17(199), 2016. ISSN 1471-2164. doi: 10.1186/s12864-016-2539-z. URL `http://dx.doi.org/10.1186/s12864-016-2539-z`.
